# The Evolution of Safe and Effective Coaguligands for Vascular Targeting and Precision Thrombosis of Solid Tumors and Vascular Malformations

**DOI:** 10.3390/biomedicines9070776

**Published:** 2021-07-04

**Authors:** Fahimeh Faqihi, Marcus A. Stoodley, Lucinda S. McRobb

**Affiliations:** Department of Clinical Medicine, Faculty of Medicine, Health and Human Sciences, Macquarie University, Sydney, NSW 2109, Australia; fahimeh.faqihi@hdr.mq.edu.au (F.F.); marcus.stoodley@mq.edu.au (M.A.S.)

**Keywords:** brain arteriovenous malformations, radiation, targeted therapy, thrombosis, tumor, vascular malformations, vascular targeting

## Abstract

In cardiovascular and cerebrovascular biology, control of thrombosis and the coagulation cascade in ischemic stroke, myocardial infarction, and other coagulopathies is the focus of significant research around the world. Ischemic stroke remains one of the largest causes of death and disability in developed countries. Preventing thrombosis and protecting vessel patency is the primary goal. However, utilization of the body’s natural coagulation cascades as an approach for targeted destruction of abnormal, disease-associated vessels and tissues has been increasing over the last 30 years. This vascular targeting approach, often termed “vascular infarction”, describes the deliberate, targeted delivery of a thrombogenic effector to diseased blood vessels with the aim to induce localized activation of the coagulation cascade and stable thrombus formation, leading to vessel occlusion and ablation. As systemic delivery of pro-thrombotic agents may cause consternation amongst traditional stroke researchers, proponents of the approach must suitably establish both efficacy and safety to take this field forward. In this review, we describe the evolution of this field and, with a focus on thrombogenic effectors, summarize the current literature with respect to emerging trends in “coaguligand” development, in targeted tumor vessel destruction, and in expansion of the approach to the treatment of brain vascular malformations.

## 1. Introduction—The Origins of Vascular Targeting for Precision Thrombosis

Vascular targeting can be broadly described as any approach that aims to cause the destruction or occlusion of abnormal or pathological vasculature. In its original context, it was used to describe the ablation of tumor vasculature, and originated from the early recognition of several important observations: (1) the penetration of therapeutic antibodies into solid tumors is poor; (2) the tumor vasculature plays an essential role in the growth and survival of cells within solid tumors; and (3) the endothelium of tumor vasculature is molecularly and phenotypically distinct from normal, healthy vasculature, making it a targetable entity [1,2]. It was thus hypothesized that targeting the accessible vasculature, rather than tumor cells themselves, was a valid alternative approach to tumor destruction [3,4,5,6].

Early hypotheses touted interruption of tumor angiogenesis as a means to prevent tumor growth and metastasis [7,8]. Multiple clinical applications of anti-angiogenic therapies have since ensued, with varying degrees of success [9]. While this technique can inhibit new vessel formation, it lacks the ability to destroy established vasculature, eventually allowing tumor cells to evolve and escape [10]. Denekamp was the first to hypothesize that the proliferating tumor vasculature should be molecularly distinct from the normal vasculature, and thereby allow targeted delivery of toxic agents to break down the existing vasculature and induce tumor necrosis [11]. Numerous molecules expressed exclusively at the surface of tumor endothelium have since been identified [12,13]. However, while the use of targeted agents increased selectivity, the issue of off-target toxicity related to the delivery of cytotoxic agents still remained [14,15].

**Figure 1:**
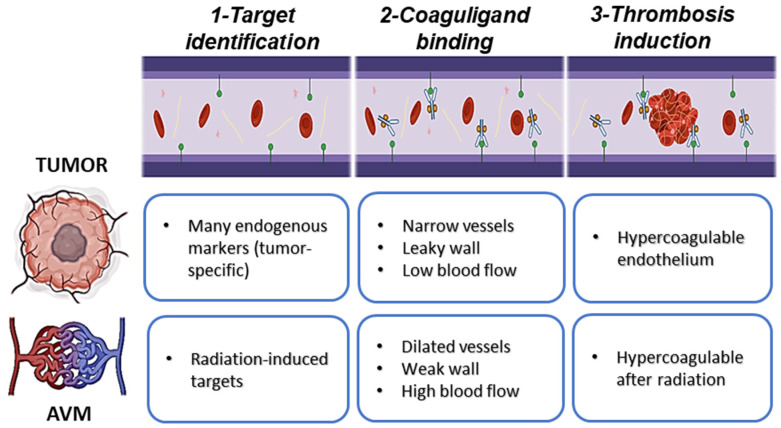
Vascular targeting with precision thrombosis in tumor and brain arteriovenous malformations (AVMs). Vascular targeting with precision thrombosis has potential for application in solid tumor destruction and occlusion of rupture-prone brain AVMs. In vascular targeting, a molecule uniquely expressed at the luminal surface of the diseased vasculature is identified as a target (1); a ligand (e.g., antibody, aptamer, peptide, or protein) with binding affinity for the target is attached to a thrombotic effector (e.g., thrombin, truncated tissue factor, or truncated staphylocoagulase) to form a “coaguligand” and delivered in the bloodstream (2); accumulation of the coaguligand at the target site in the presence of an inherent or induced hypercoagulable endothelial surface induces thrombus formation, leading to vessel occlusion (3). Adapting this approach for each pathology has its own challenges. Endogenously expressed surface markers which discriminate the endothelium of brain AVMs remain unknown. Stereotactic delivery of ionizing radiation to the AVM volume can be used to stimulate novel markers at the endothelial surface for both AVMs and tumor vasculature. Radiation also stimulates a procoagulatory surface in brain AVMs and enhances it in tumor vasculature. The large, dilated vessels in AVMs present extra challenges in the approach: high flow and shear stress may require high-affinity target-ligand interactions and effectors with greater thrombogenic potential. In tumor targeting, tumor cells at the rim may evade destruction. Vascular destruction is not required for AVMs however occlusion must be complete (all vessels stably thrombosed). [Original figure, created with BioRender.com by F. Faqihi].

It was P.E. Thorpe who first conceived the notion of targeted thrombosis and the use of “coaguligands” to limit this potential toxicity [3,4,16,17]. Coaguligands couple a targeting ligand (that can recognize a specific molecule on the endothelial surface) with a thrombogenic effector that can induce the coagulation cascade (Figure 1). Through binding in a localized fashion to the inherently pro-coagulatory surface of tumor endothelium, and accumulating to a threshold level, rapid occlusion of the pathological vessels could be obtained (vascular infarction), essentially starving the fast-growing tumor of nutrient and oxygen supply, leading to tumor necrosis. In the last 25 years, the field has continued to evolve, with multiple groups investigating iterations of this approach for tumor vascular targeting in various malignancies, and here we review all pre-clinical studies published in this time, up to the most recent human safety trials.

Vascular infarction as an approach may also be suitable for re-purposing in the treatment of brain vascular malformations. Brain arteriovenous malformations (AVMs) are a disorder in which inherited germline mutations or sporadic mosaic mutations in the endothelium disrupt normal vessel development and maturation, resulting in the formation of arterio–venous connections that lack an interconnecting capillary bed (Figure 1) [18,19,20,21]. Initial high arterial forces cause massive dilation of the draining veins, leading to the development of relatively weak-walled arterialized veins which are highly prone to the risk of rupture [22]. While AVMs can occur anywhere in the body, when they occur in the brain they are a major cause of intracerebral hemorrhage, particularly in children and young adults [23]. Vascular targeting with precision thrombosis could be highly applicable to the treatment of AVMs, and potentially other vascular malformations such as cavernous malformations, as the abnormal vessels do not supply blood to the brain, but instead act as a vascular shunt that pushes blood flow across the brain, often depriving the surrounding brain of adequate perfusion [24]. For this reason, complete removal or occlusion of these vessels is necessary to enact cures and remove the risk of rupture. Surgical removal or slow occlusion by stereotactic radiosurgery are used currently, however these approaches carry inherent risks, or a degree of inefficacy, and are often unsuitable for those patients with large (>3 cm diameter) or deep AVMs [25]. There remains a high, unmet need for a non-invasive pharmacological approach to AVM treatment. However, translation of the vascular infarction approach for treatment of intractable AVMs is not without its own challenges. Firstly, at ~2 mm in diameter, the dilated AVM vessels are considerably larger than tumor vessels (the latter being typically capillary in nature), and secondly, AVM vessels endure higher flow rates and increased shear stress [24]. Thus, consideration must be given to ligand binding affinity and thrombus stability so that the thrombosis induced in AVMs is substantial and robust. Secondly, AVM vasculature is poorly characterized at the molecular level, and native surface biomarkers that can discriminate the diseased endothelium have not yet been identified. In addition, targetable markers must be identified on the arterialized vein, but not en passant feeding vessels. We discuss how priming of the AVM vasculature using focused stereotactic radiosurgery is being explored to overcome some of these challenges, as an approach that can induce, not only a pro-coagulatory surface, but potentially unique discriminatory targets (Figure 1).

Part I of this review focuses on the vascular markers and associated coaguligands investigated to date in animal models of tumor or AVM vascular infarction, and the first human trials. In Part II, the review focuses on other methodological modifications that aim to increase the efficacy of the approach, with a more focused discussion on thrombogenic effectors and their safety.

## 2. Part I—Vascular Targets: Balancing Specificity against Efficacy

Since Denekamp first verbalized the recognition that tumor vasculature should be molecularly different from normal vasculature, and hence targetable [11], many up-regulated endothelial targets have been identified in association with various tumor types. A limited number of these have been tested specifically in the context of tumor infarction models (Table S1). To selectively induce vascular thrombosis, an endothelium-specific molecular signature is crucial. A prospective molecule, whether induced or naturally existing, must be prominently present at the luminal cell surface of diseased endothelial cells, and must be absent, or at least minimally expressed, on the luminal surface of healthy vessels. In contrast to cytotoxic drug delivery, off-target expression on epithelial tissues or other external surfaces is not a major concern for delivery of thrombotic agents in this approach, as they do not contact coagulation factors in the blood. Similarly, the rapid endocytosis and internalization, often necessary for cytotoxic drug actions, is not required for vascular infarction. In fact, rapid intracellular translocation is undesirable as extended retention of the surface receptor, and its bound coaguligand at the luminal surface is more favorable for sustained engagement of the coagulation system. Here, we review the succession of markers examined in multiple animal models to date and summarize key information in Table S1.

### 2.1. MHCII

The first studied vascular marker was MHCII. The Thorpe laboratory produced the first bi-functional agents conjugating an antibody targeting experimentally induced MHCII with truncated tissue factor (tTF) as the thrombogen [16]. Intravascular administration to mice with large neuroblastomas successfully induced selective thrombosis, resulting in complete tumor regression in 38% of the mice tested. While this early experiment did not use an endogenous marker of the tumor endothelium, this study gave the first proof-of principle that thrombosis and infarction could be induced selectively in tumor vessels by targeted delivery of a thrombogen to a discriminatory marker *not* normally expressed on the healthy endothelium.

### 2.2. VCAM-1

Thorpe et al. then moved onto investigation of endogenous targets, of which the first was vascular cell adhesion molecule 1 (VCAM-1/CD106), a cytokine-inducible inflammatory surface molecule expressed by the tumor vasculature and some tumor cells. Using a mouse model of Hodgkin’s lymphoma, antibody-directed targeting of tTF to VCAM-1 obliterated some vessels, reducing mean tumor volume by 45% after 3 weeks, but was not enough to entirely kill the tumors [17]. Dienst et al. later tested the anti-tumor activity of a recombinant fusion protein targeting tTF to both murine and human VCAM-1 antigens, using three human xenograft models (L540rec Hodgkin lymphoma; Colo677 small-cell lung carcinoma; and Colo677/HDMEC small-cell lung carcinoma with human vasculature) [26]. Coaguligands were co-administered with lipopolysaccharide (LPS) to enhance VCAM-1 expression and doxorubicin (Dox) to sensitize cells to coagulation induction. Outcomes varied between models and treatment regimens, with necrosis rates between 26–74%. Delayed tumor growth and coagulation were observed in the tumor vasculature without off-target thrombosis, however tumor eradication was similarly incomplete (14–30%). Interestingly, it was observed in both these studies that these VCAM-1-targeting coaguligands demonstrated a high degree of off-target binding in venules of the lung and heart. However, this occurred without any histochemically visible thrombosis [26]. Rather than attributing the poor tumor eradication to suboptimal target expression, the authors’ hypothesized that the high level of off-target binding caused overt coaguligand absorption, reducing accumulation within the tumor volume. This early finding highlighted two important aspects. Firstly, that the inherently hypercoaguable surface of tumor endothelium relative to normal endothelium [27] supplies other molecular factors essential for infarction, apart from the expressed target, that cooperate locally. This characteristic need for local cofactors presented on the diseased vessels contributes significantly to the specificity and safety of this approach relative to cytotoxic drug delivery. Secondly, these studies suggest that, from a safety perspective, disseminated expression of a molecule may not exclude investigation of its potential as a target, but could contribute to poor efficacy due to reduced coaguligand accumulation at the target site.

### 2.3. Phosphatidylserine (PS)

Thorpe hypothesized from the latter studies that restriction of thrombosis to the tumor volume was a result of coincident exposure of phosphatidylserine (PS) at the surface of the tumor endothelium [17,28,29]. PS is a phospholipid component of the plasma membrane that, in normal, healthy cells, faces the intracellular cytoplasmic compartment and is not accessible to the circulation [30]. In response to various stimuli, however, PS can be relocated to the external leaflet, exposing it to blood components [31]. PS exposure is most commonly associated with apoptosis and is used extensively as an apoptotic marker [32,33], but also occurs in non-apoptotic tumor cells where it contributes an immune- and inflammation-dampening role [31]. PS exposure also plays an essential role in activation of the extrinsic coagulation pathway. Its electrostatic charge attracts clotting factors in the blood, but its interaction with TF at the cell surface is essential for pro-thrombin activation [31]. Hence, targeting of TF in the absence of PS exposure, that is, normal healthy vasculature, limits thrombus formation.

Thorpe’s observations regarding PS and the pro-coagulant nature of the tumor surface was not only important from a safety perspective, but led to PS itself being investigated as a potential vascular target. It was theorized by the group that PS exposure across a broad variety of tumor types could allow it to act as a pan-cancer target with the development of a one-drug-fits-all approach. Subsequent imaging studies successfully showed that use of the PS-targeting protein ligand, Annexin V, recognized the externalized PS preferentially on the luminal surface of tumor-associated vessels in mice bearing a series of solid tumors [28]. However, when an Annexin V-tTF chimera was used to target PS in a vascular injury model, the effects could be either pro- or anti-coagulatory, depending on the dose; high dose Annexin V binding essentially neutralizing the cofactor actions of PS for TF-dependent Factor VII interaction and activation [34]. These studies highlight that consideration must be given to how potential target-ligand-effector interactions may ultimately affect the balance of the coagulation cascade.

While PS targeting in tumors has been more avidly pursued in the context of cancer immunotherapy [35], it has been investigated as a radiation-stimulated target for vascular infarction in brain AVMs [36,37,38,39,40]. Thorpe and colleagues demonstrated that translocation of this phospholipid to the surface of tumor vessels could be enhanced in response to oxidative stress, via stimulation by growth factors, hydrogen peroxide, and ionizing radiation [28,41,42]. This raised the possibility that PS could be used as a prospective, feasible marker in combination with a PS inducer in tumors or in AVMs. A further benefit of radiation as a vascular primer is its ability to induce a pro-coagulatory state at the endothelial surface (e.g., down-regulation of thrombomodulin, up-regulation of platelet adhesion molecules) that shifts the local milieu to support thrombosis induction, similar to that found on tumor vasculature [43,44,45]. PS targeting was first investigated in an extracranial rat AVM model using a ligand-independent approach [36,37]. Non-targeted, soluble tTF and lipopolysaccharide were administered after delivery of a clinically relevant dose (25 Gy) of ionizing radiation to a surgically created arterio-venous fistula. While only small vessels within the AVM nidus were occluded, the thrombi were durable without off-target coagulation in non-irradiated vessels or other organs. Subsequent studies then used a ligand-directed approach to improve targeted occlusion of the larger AVM vessels. Using a PS-targeting annexin V protein conjugated to thrombin as an effector, it was first established in vitro that rapid and selective thrombosis could be induced on an irradiated endothelial cell layer using whole human blood circulating in a parallel-plate flow system [40]. In vivo studies subsequently demonstrated that significant occlusion of the rat model AVM vessels could be achieved with this coaguligand [46]. Although, in the latter study, both non-irradiated and irradiated AVMs showed thrombotic occlusion at high conjugate doses (potentially due to altered hemodynamics in the surgically created AVM [47]), specificity at the irradiated tissue was improved by coaguligand dose reduction, although with a concomitant reduction in efficacy [46]. These foundational studies using PS as the molecular target provided proof-of-principle that vascular infarction could be applied to the larger high flow vessels of brain AVMs using a radiation-priming approach for specific target induction as well as induction of a supportive pro-coagulatory endothelial surface.

### 2.4. Vascular Endothelial Growth Factor (VEGF) Receptors

VEGF plays a vital role in tumor neovascularization and the overexpression of VEGF isoforms, and their receptors have been well studied in tumor neovasculature [48,49,50,51]. VEGF remains a primary target in anti-angiogenic treatment strategies using anti-VEGF therapies such as Bevacizumab (Avastin^®^), Ranibizumab (Lucentis^®^), and Pegaptanib (Macugen^®^) [52,53,54]. For vascular infarction, various ligands targeting the associated surface-bound VEGF receptors have been investigated. In 2005 for example, El-Sheikh et al. used intravital microscopy to examine microvascular thrombosis in the context of colon, mammary, prostate, and lung carcinoma after infusion of a tTF-fused coaguligand consisting of the heparin binding domain of VEGF165 (HBDt, exon 7) that recognizes a trimolecular complex of VEGFR-2 (KDR/Flk-1/CD309), the VEGF165 co-receptor, neuropilin-1 (NRP-1), and the proteoglycan, chondroitin C sulfate (C6S) [55]. Relatively poor thrombotic induction was observed with the single agent, however an 80% reduction in conjugate dose requirement was achieved by co-infusion of human VIIa, a factor assumed to be limiting in the animal model. Similarly, Huang et al. conjugated the natural VEGF ligand to engineered tTF, and achieved limited infarction in colon carcinoma xenografts in 20% of animals after two doses [56]. More recently, Lv et al. used a VEGFR-1 (Flt-1)-targeting peptide called SP5.2 (NGYEIEWYSWVTHGMY) fused with tTF in Balb/c mice harboring sarcoma 180 and reported 70% tumor inhibition [57]. Multiple ligands have been used to target the NRP-1 co-receptor in the context of tumor vascular thrombosis. Three recent studies effectively targeted tTF to NRP-1 using a monoclonal antibody and a bi-cyclic polypeptide (tTF-EG3287) that targets the binding site of VEGF165 to NRP1 [58,59,60]. In human liver and colorectal adenocarcinoma models, these agents demonstrated high binding affinities and significant inhibition of tumor growth without off-target effects.

### 2.5. Integrins and Integrin Receptors

Integrins are receptors that allow cell attachment to either the substratum or to adjacent cells. On the endothelium, they are typically localized to the basolateral surfaces and inaccessible to blood in the lumen. In tumors, the vasculature is typically poorly formed and leaky, and frequent cell detachment can expose these molecules to the blood flow [54]. In addition, both tumor cells and tumor endothelium up-regulate integrin expression [61,62]. Integrin receptors are also expressed in endothelial progenitors and tumor neogenic vasculature but are absent or sporadically expressed in healthy adult vessels [63]. Fernando et al. used a tTF-fused antibody-conjugate, targeting the integrin receptor TEM8 (tumor endothelial marker 8), to disrupt tumor vessels in a xenograft model of human colorectal carcinoma and found high targeting specificity allowing inhibition of tumor growth but without necrosis [64].

The most frequently assessed integrin-targeting peptides for use in tumor vascular targeting, however, are RGD (arginine-glycine-asparagine; GRGDSP) and NGR (asparagine-glycine-arginine; GNGRAHA). Both peptides derive from an amino acid sequence found within the matrix protein, fibronectin [65,66]. RGD is the most common peptide motif mediating attachment of cells to the extracellular matrix, primarily via alpha(v)beta(3) integrin (α_v_β_3_) [67]. The second peptide, NGR, primarily targets the membrane-bound metalloprotease, aminopeptidase N (APN or CD13) in particular, an isoform expressed predominantly on angiogenic endothelium, normally associated with tumors [68,69,70]. NGR also shows affinity for α_v_β_3_ after spontaneous deamidation of its asparagine group to produce isoDGR [71]. The dual targeting potential of NGR was hypothesized to allow more consistent surface coverage relative to RGD, enhancing the ability to reach coagulation thresholds [72], and to allow targeting of larger tumor vessels which was shown to be limited when targeting α_v_β_3_ alone [73].

An early study by Liu et al. demonstrated selective thrombosis in a colon carcinoma mouse model using tTF fused to multiple fibronectin type III repeat units containing RGD [74]. In the same model, Huang et al. later demonstrated induction of significant intravascular thrombosis and tumor necrosis, tumor growth inhibition, and increased survival using a construct with three RGD repeats fused to tTF [75]. Multiple other studies have investigated the efficacy of RGD and NGR-targeted coaguligands, primarily fused to the C-terminal region of tTF, with pre-clinical testing performed in human breast cancer (SKBR3 and MDA-MB-435), lung adenocarcinoma (A549 and MAD109), human fibrosarcoma (HT1080), glioblastoma (U87), melanoma (M21), small cell lung cancer (HTB119), and colorectal (CT26) cancer models (Table S1) [14,76,77,78,79,80,81,82,83]. A study by Hallahan et al. used embolic nanoparticles rather than tTF to target integrin α2bβ3 on radiation-primed tumor vessels [84]. This integrin is normally expressed on activated platelets but after radiation platelet accumulation at the endothelial surface allowed vessel targeting with RGD peptides or fibrinogen. Thrombosis was confirmed with 90% reduction in tumor blood flow in a Lewis Lung carcinoma model.

All studies indicated that tTF-ligand constructs demonstrate good specificity without significant off-target thrombosis with intravenous injection doses < 5 mg/kg, although the efficacy was study specific. Greater efficacy was typically demonstrated in highly vascularized tumors with regard to growth inhibition, induction of necrosis, and survival. Complete tumor regression was limited, and regrowth tended to occur from cells surviving at the tumor rim. Early attempts at subcutaneous injection of NGR-tTF (1–5 mg/kg) demonstrated low anti-tumor activity and also significant toxicity [14]. However, recent studies suggest repeated lower doses are more tolerable and effective, however repeated intravenous injections given by slow infusion remain superior with respect to efficacy [82]. Other recent studies have reapproached these ligands using alternative thrombogens for coaguligand construction, with evidence of complete tumor infarction observed in mouse mammary and human prostate xenografts by delivering truncated coagulase (tCoa) bearing an RGD or NGR motif [85,86].

### 2.6. Matrix Targeting

While most targets investigated are typically ligands or receptors, normally associated with the endothelial surface, the greater accessibility of underlying extracellular matrix or basement membrane in poorly developed tumor vasculature means they can be targeted from the circulation, and allow vascular infarction due to their ready accessibility to blood components. Fibronectin (FBN) is an extracellular matrix protein produced by all cells in the body to which various integrins bind [87]. There are, however, >20 different isoforms of this protein that are produced under different conditions, or by different cell types, the functions of which are not particularly well understood. One isoform is the result of alternative pre-mRNA splicing that retains an intron in the mRNA transcript resulting in expression of a protein with an extra 90 amino acid epitope called the extra-domain B (EDB) [88]. This FBN isoform is an abundant molecule in the matrix of many aggressive solid tumors and contributes to vasculogenesis in embryos and angiogenesis in cancer [89]. The relative absence of this isoform in the normal vasculature makes it a putative marker for vascular targeting. Nilsson et al. tested a specific antibody fragment (ScV) targeting the EDB domain in three types of solid tumors (murine teratocarcinoma, murine colon adenocarcinoma, and rat fibroblast). In these mouse models, tTF fusion to ScV resulted in complete tumor eradication in 30% of the treated animals without apparent side effects [90]. Hu et al. also targeted fibronectin with a recombinant coaguligand (hTV-1-tTF) but found limited efficacy in medium to large tumor vessels [73]. However, a second coaguligand (hTNT-3-tTF) targeting DNA exposed in degenerative areas of these tumors achieved greater success in the same study.

Rather than targeting fibronectin directly, a recent adaptation of the approach by Shi et al. delivered TF to microthrombus-associated fibrin-fibronectin complexes overexpressed on tumor vessel endothelium and tumor matrix [91]. A peptide (CREKA) identified by phage display was fused to tTF, and found to be effective at causing intratumoral thrombosis and inhibition of tumor growth in hepatocellular colon and breast cancer models at significantly lower doses relative to other thrombogenic constructs. The tTF-CREKA molecule is hypothesized to have self-amplifying thrombogenesis due to its ability to recognize both original microthrombi complexes present at degenerative tumor sites, as well as recognize new clotting-associated fibronectin-fibrin complexes which form as the first coaguligands bind. Most recently, the CREKA peptide was used to target nanoparticles delivering thrombin in breast and melanoma mouse models, as well as mouse and rabbit liver cancer models [92]. Moderate effects on tumor growth were observed with thrombin delivery alone, however combination with the chemotherapeutic agent doxorubicin induced complete regression in some models.

Another novel approach utilized the inherent acidic extracellular pH of the tumor microenviroment [93] to allow selective binding of recently developed recombinant coaguligand, tTF-pHLIP [94]. Extracellular acidification, caused primarily by anaerobic metabolism and lactate secretion, allows conformational changes to the “pH (low) insertion peptide” (pHLIP) to form a transmembrane alpha-helix that can insert into the cell membrane within the acidic environment. This pH-responsive fusion protein was shown to selectively induce tumor infarction, delay growth in human breast xenografts [94], and, more recently, to induce growth reduction and tumor shrinkage in a mouse melanoma model with just 2.5–5 μg of tTF fusion protein [95].

Brand et al. recently assessed the anti-tumor activity of two peptide-guided fusion proteins (tTF-TAA and tTF-LTL) targeting a surface proteoglycan called NG2, exposed on angiogenic pericytes in lung adenocarcinoma xenografts. The tTF-TAA fusion protein was found to reproducibly induce vascular occlusion and growth inhibition, however, relative to tTF-NGR, activity was low with a small therapeutic window [96].

### 2.7. Translocated Intracellular Proteins

Several biomarkers of the tumor vasculature include proteins that are traditionally located intracellularly, but appear to translocate to the cell surface in the disease context. These markers have high potential for discrimination and selectivity given their absence at the cell surface in the normal healthy endothelium. In 2002, Liu et al. targeted prostate-specific membrane antigen (PSMA) with an antibody linked to tTF in a Mat Lu prostate tumor model [97]. This type 2 transmembrane glycoprotein is normally found intracellularly, but becomes surface distributed in transformed prostate epithelial cells and, through vascular mimicry and formation of a pseudoendothelial phenotype, this marker becomes targetable on the intertumoral vasculature [98]. This coaguligand achieved 70% selective tumor thrombosis immediately after administration [97]. The uniqueness of this target allowed great targeting specificity, however heterogeneous expression of this marker between vessels (true endothelium versus endothelial mimics) limited efficacy.

In recent experiments by Li et al., externalized nucleolin was used as a vascular target [99]. Nucleolin is a protein that exists typically in the nucleus or nucleolus, but its translocation to the cell membrane in angiogenic associated-tumor vessels has been observed [100]. Li et al. targeted nucleolin using a “DNA nanobot”, consisting of cross-linked sheets of DNA functionalized with nucleolin-targeting DNA aptamers, designed to deliver active thrombin encased in a central channel. The thrombin becomes exposed only at the relevant site upon contact of the nanobot with nucleolin at the endothelial surface. This nanobot successfully occluded tumor vasculature with moderate growth inhibition in moderately vascularized mouse models of human breast cancer and human ovarian cancer. Complete regression was achieved in 35% of animals in a highly vascularized model of melanoma (B16-F10) [92,99].

Translocated intracellular proteins have also been investigated as radiation-induced targets in brain AVM models. Several intracellular proteins, such as the molecular chaperone, alpha-B-crystallin (CRYAB), and mitochondrial proteins such as the E2 subunit of pyruvate dehydrogenase (PDCE2/DLAT), have been shown to be externalized on the endothelium in response to radiation [101,102,103]. Preliminary studies with CRYAB-targeting antibodies conjugated to thrombin have shown selective binding to irradiated human endothelial layers and dose-dependent thrombus induction in vitro using a parallel-plate flow system and whole human blood [104]. Radiation stimulation of unique targets not normally present on the healthy endothelium, together with induction of a hypercoaguable state in the target zone, promises a highly selective approach to treatment if in vivo target expression and surface distribution are sufficient to support effective occlusion of large AVM vessels.

### 2.8. Targets in Human Translation

A tTF-NGR construct is the only agent to be taken to Phase I clinical studies to date. The NGR homing peptide is also in clinical trials as a ligand for targeted cytokine delivery [70,105]. Bieker et al. reported the first in-man experience for vascular infarction using administration of low dose tTF-NGR for late stage metastatic solid tumors (cholangiocarcinoma and adenocarcinoma), and demonstrated reduced tumor perfusion and good tolerance [106]. Recently, Schliemann et al. performed a Phase I dose-escalation study using tTF-NGR in 17 patients with advanced malignant cancers, again demonstrating the ability to reduce tumor perfusion with good overall tolerability [107]. Daily infusion over a 1 h period for 5 days, followed by a 2-week rest prior to a subsequent cycle with dose escalation, established a maximum dose tolerance of 3 mg/m^2^/day. Strong and selective decreases in lesion blood flow of up to 90% were observed in metastatic lesions of the liver, however without extended patient survival. Off-target effects included thromboembolic events associated with catheter delivery, deep vein thrombosis, and detection of high sensitivity troponin T, however no events were life-threatening and all could be reversed with anti-coagulant administration.

### 2.9. Summary—Part I

The broad conclusions drawn from examining the literature around vascular targets used for infarction studies is that specificity appears readily achievable using this vascular targeting approach, in both tumor models and in vascular malformations, however efficacy is difficult to achieve consistently. The most promising and consistent coaguligands in tumor targeting to date have utilized the dual targeting NGR and self-amplifying CREKA peptide, however it is somewhat difficult to compare target efficacy with the use of multiple different ligands and procoagulant combinations, across multiple cancer models. Human studies with tTF conjugated to NGR demonstrate good tolerability but, to date, trials in advanced cancer patients have not yet demonstrated the human efficacy (that is, increased survival), as may be expected in this type of cohort and a Phase I trial. Hence, studies that continue to investigate alternative targets or modified approaches for amplifying tumor infarction remain important. While the feasibility of large vessel occlusion has been established in AVM models using thrombin, the primary focus of current research in this disease remains establishing valid discriminatory targets, either with or without radiation priming. Investigation of alternate thrombogenic effectors with greater efficacy and/or safety profiles remains important, and in Part II we examine in greater detail how thrombogenic effector choice can potentially affect both efficacy and safety.

## 3. Part II—The Importance of Thrombogenic Effectors in Precision Thrombosis

**Figure 2:**
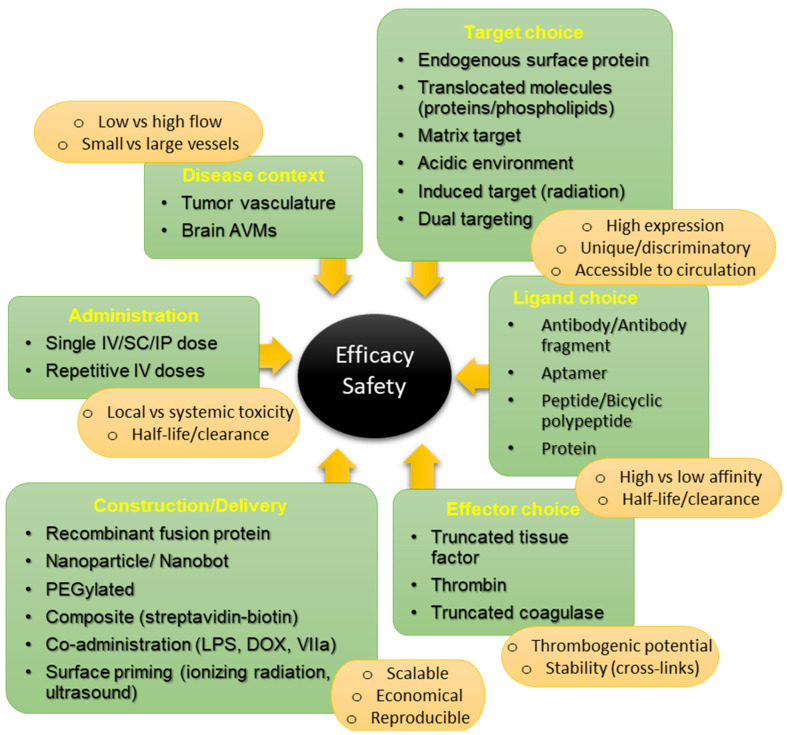
Considerations in coaguligand development that influence efficacy and safety. In the field of vascular targeting with precision thrombosis, many factors must be considered in the choice of coaguligands that achieve substantive efficacy without compromising safety in each disease context. Many endogenous targets have been investigated for selectivity in the context of solid tumor targeting, but expression levels are still considered a primary limiting factor for complete tumor ablation. Ligand affinity, as well as rate of clearance, may influence binding under high or low flow and subsequent efficacy within the therapeutic window. In particular, the choice of effector may play a significant role in efficacy for large versus small vessels, depending on individual thrombogenic potential. This must be balanced with stability of clot formation, and any risk of emboli or disseminated intravascular coagulation. Novel approaches to coaguligand construction or delivery that increase effector activity without exceeding toxic thresholds continue to be investigated. [Original figure, created by F. Faqihi].

Most authors agree that efficacy in vascular infarction is primarily dependent on target expression and distribution at the surface, factors that limit the ability of thrombogenic effectors to accumulate locally and surpass anti-coagulation thresholds. As target expression levels may be difficult to control, manipulation of other factors must also be considered to achieve the desired goals. Figure 2 summarizes interacting factors that can influence efficacy and safety in vascular infarction. Here, we consider thrombogenic effectors in greater depth, considering the specific properties that each brings to precision thrombosis that influence their use, and outline studies that describe novel approaches to increase thrombogenic potency without exceeding toxic thresholds that lead to disseminated intravascular coagulation (DIC).

**Figure 3:**
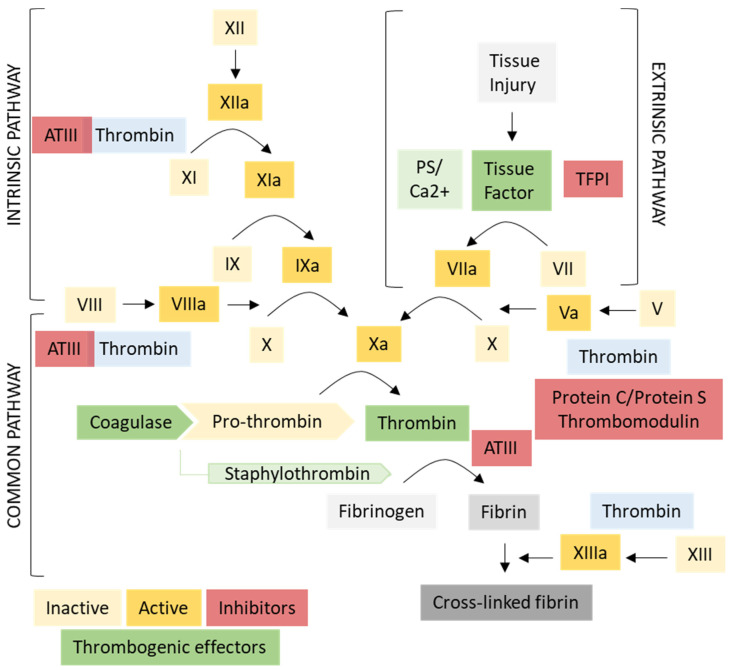
Thrombogenic effectors within the coagulation pathway. The three thrombogenic effectors used in vascular infraction studies (green) enter the coagulation pathway at different points, influencing their potential efficacy and safety. Use of tissue factor (TF) as coaguligand effector requires interaction with exposed phosphatidylserine (PS) on activated surfaces such as tumor or irradiated endothelium. This requirement limits the possibility of systemic clotting, producing a higher safety profile, but also limits coagulation efficacy. Thrombin lies in the common pathway directly cleaving fibrinogen to fibrin, and removes the requirement for early enzymatic steps that are subject to feed-forward and feedback mechanisms (e.g., TFPI = tissue factor pathway inhibitor). Thrombomodulin expression is lowered on tumor endothelium, and after radiation, thus removing protein C/S activation and Factor V inhibition. Thrombin amplifies the cascade through activation of Factors XI, VIII, and V, but also activates the fibrin-stabilizing factor XIII to produce stable cross-linked clots. Coagulase from *Staphylococcus* forms a complex with pro-thrombin called staphylothrombin which cleaves fibrinogen exclusively. This complex does not activate XI, VIII, or V as native thrombin does, but is resistant to inactivation by anti-thrombin III (ATIII) and has high thrombogenic potential. Coagulase does not activate XIII, so cross-linking and clot stability must be assessed in future studies. [Original figure created by L. McRobb and F. Faqihi].

Three types of thrombogenic effector molecules have been explored in vascular targeting studies to date: tTF, truncated coagulase (tCoa), and thrombin. Each agent acts at a unique stage within the coagulation cascade, requiring the presence of distinct cofactors or the absence of inherent inhibitors for optimal activity (Figure 3). Depending on these factors, each agent may need to reach different threshold concentrations for thrombosis induction to occur, which will influence both efficacy and safety. We will address each of these agents with respect to their localization in the coagulation pathway, their contribution to selectivity (safety), as well as examine their use and efficacy to date in various pathological contexts.

### 3.1. Tissue Factor (TF)

Tissue factor (TF) was the first coagulation effector used in vascular targeting for proof-of-principle and remains the most widely published to date in vascular infarction studies (Table S1). Recombinant tTF was shown to be readily and economically produced in the laboratory [108], and readily deliverable to the endothelial surface by conjugation to antibodies or by fusion with homing peptides [16,17,106]. TF is an integral membrane protein present in the endothelial and smooth muscle cell layers of the vascular wall, and is the key instigator of the extrinsic coagulation cascade (Figure 3) [109]. On the normal endothelium, TF is poorly expressed, however in response to various stimuli, TF is translocated rapidly to the cell surface [110]. Exposure at the surface allows interaction with circulating Factor VII, creating a functional active complex (TF-VIIa) and producing a series of enzymatic reactions (IX, X, and pro-thrombin activation) that culminate in the common coagulation pathways and cleavage of soluble fibrinogen in the blood to produce insoluble, polymerized, and cross-linked fibrin deposits and clot formation [111].

Structurally, the TF protein has a hydrophobic membrane domain that anchors it to the cell, and a soluble, external domain that is responsible for the binding and enzymatic conversion of Factor VII in the blood. This N-terminal domain (amino acids 1–219) represents truncated TF (tTF) [112]. While in the circulation, this soluble fraction has little coagulant activity until it interacts with a suitable pro-coagulant surface, most notably requiring close proximity to exposed cellular PS, which acts as a co-factor [34,113]. This requirement confers tTF with a rather favorable safety profile, as demonstrated by Thorpe and collaborators when off-target binding of a VCAM-1 targeting coaguligand in the lung was not followed by off-target thrombosis [17], something that could not be achieved if delivering a freely active, cytotoxic drug.

A major benefit of tTF use has been the ability to create recombinant plasmids that fuse homing peptide sequences at its N-terminus. Removal of the membrane-bound domain increases solubility, allowing delivery through the blood, but also allows fusion of a homing peptide in a controlled way that allows directional exposure of tTF to the blood stream. That is, while the homing ligand attaches at the cell surface, the TF tail extends into the blood flow to capture circulating Factor VII. This approach allows reproducibility in coaguligand construction, as there is no requirement for any secondary ligation between ligand and effector, so the molecules are bound in a defined way without loss of activity. This purely recombinant approach is economical and scalable, avoids the requirement for humanization of antibodies, and limits the probability of immune reactivity [78].

In animal models, delivery is predominantly via intravenous (i.v.) injection, with a therapeutic window for tTF constructs established within a dose range of 1–4 mg/kg (up to 35 μg i.v. in mice). Attempts at subcutaneous application with tTF conjugates in pre-clinical studies have been associated with lower efficacy and more significant toxicity [14]. Primary concerns with i.v. delivery relate to observations of incomplete thrombosis and tumor cell survival associated with limited target expression rather than toxicity. Attempts to overcome incomplete anti-tumor activity with delivery of higher doses of TF constructs (>5 mg/kg) has resulted in systemic toxicity in these animal models [114].

### 3.2. Approaches to Increase TF Coaguligand Efficacy

The systemic toxicity observed with higher doses of tTF places a limit on the extent to which efficacy could be improved by dose escalation alone, although repeat administration at lower doses is promising [83,107,114] (Table S1). As stated, limited efficacy is thought primarily to lie with levels of target expression and surface coverage, as this dictates how much thrombogenic effector can be localized to overcome coagulation thresholds. Other approaches to increase tTF localization and activity without increasing dose in some mouse models include co-administration of Factor VIIa to enhance activity and reduce coaguligand dose, as this is thought to be limiting in mice [55,97]. However, how this relates to efficacy in humans is unclear.

Dual targeting is an obvious approach to increase localization that is already reflected in the superior performance of ligands that recognize more than one target, as described in Part I for the NGR homing peptide which targets both α_v_β_3_ and CD13. Simultaneously targeting two independent biomarkers would be expected to increase coverage of the endothelial surface to attain coagulation thresholds. Using this concept, Thoreau et al. conjugated two targeting ligands, an RGD peptide (cyclic RGDyK) recognizing CD51 and a heptapeptide (ATWLPPR) recognizing NRP-1, and used imaging to demonstrate enhanced tumor-targeting efficiency and tumor accumulation [115]. Although they did not examine vascular infarction, this demonstrates that bi-specific or dual-targeting agents have the potential to increase pro-coagulant delivery without increasing systemic TF concentrations. Chen et al. investigated a novel form of dual targeting using both peptide-guided and magnetic field-guided delivery of embolic superparamagnetic iron oxide nanoparticles (SPIO-NPs) [116]. The particles were functionalized by cross-linking a VEGFR1-targeting peptide (SP5.2) and tTF to the particle surface and, together with an external magnetic field, demonstrated increased targeted tumor delivery relative to peptide-guided particles alone. Zou et al. similarly functionalized these nanoparticles with the NRP-1-targeting fusion protein, tTF-EG3287, and demonstrated the additive effects of magnetic co-localization in HepG2 tumors [117].

An alternative approach to increasing TF localization was recently developed by Xu et al. using a two-step coagulation or “composite” approach to promote coagulation efficiency [118]. Utilizing a primary infusion of streptavidin-bound antibody targeting NRP-1, followed by a secondary infusion of biotinylated-tTF, thrombosis was increased without the need to increase the circulating TF dose. As each tetrameric streptavidin structure can interact with four biotin molecules, this approach amplifies the original signal in contrast to the direct 1:1 interactions normally observed between a single target and a single fusion protein. Simultaneous targeting of two independent targets using matched streptavidin-tagged ligands could potentially enhance the efficacy of this approach without the need to increase corresponding biotin-tTF levels.

Other recent approaches target enhancement of TF activity at the vascular wall, rather than through enhanced localization. Brand et al. found that low energy ultrasound activation of intravascular microbubbles or priming with ionizing radiation could further stimulate basal endothelial exposure of the TF cofactor, PS, with an overall enhancement of thrombotic TF effects [119,120]. This is in line with the use of radiation as both a priming agent for unique targets in AVMs and its concomitant ability to induce a hypercoaguable endothelial surface, as described in Part I.

Several studies have routinely incorporated combinatorial use of cytotoxic drugs and tumor infarction approaches. In particular the use of doxorubicin (DOX) has been shown to enhance tumor infarction [15,97]. Stucke-Ring et al. examined combinatorial effects of DOX and tTF-NGR in melanoma and fibrosarcoma xenografts [15]. DOX administration prior to tTF-NGR coaguligand injection stimulated apoptosis, increasing PS externalization and thus enhancing the pro-coagulatory effect of targeted TF. In this case, the vascular occlusion caused by the coaguligand also contributed to increased efficacy through improved drug pharmacodynamics, as occlusion increased localized drug retention and, hence, cytotoxicity.

A study by von Maltzahn et al. described a complex use of tTF conjugates in a multistep biological cascade for amplified tumor targeting with cytotoxic drugs. In this case, localized induction of the coagulation cascade by targeted tTF (or by thermal activation of gold nanorods) was not the endpoint, but was used to facilitate the accumulation of DOX-carrying liposomes functionalized with fibrin binding (FXIII) peptides [121].

Poor efficacy with tTF has also been attributed to unfavorable pharmacodynamics and rapid clearance of coaguligands from circulation. Schwoppe et al. investigated the effect of coaguligand PEGylation as an approach to improve pharmacodynamics in tumor targeting [78]. PEG-conjugated tTF-NGR demonstrated significantly reduced thrombotic activity, but an improved therapeutic range. However, in this instance both random PEGylation and site-directed mono-PEGylation did not significantly improve therapeutic outcomes [78,79]. Stucke-Ring et al. also demonstrated reduced efficacy of tTF fusion proteins after PEGylation, however when used in combination with DOX could still achieve 75% vascular infarction, compared to 90% infarction with non-PEGylated constructs used in combination with DOX, and 45% infarction in non-PEGylated constructs without DOX [15].

In summary, use of tTF alone has not always provided complete tumor regression in animal models to date. The upper threshold for tTF toxicity may be overcome using these novel approaches that amplify local activity without systemic toxicity. Alternatively, other activators of coagulation might provide more effective solutions.

### 3.3. Thrombin

The placement of TF actions relatively early in the coagulation cascade, as well as the requirement for PS as a cofactor at the endothelial surface, are important contributors to its superior safety profile, but may also play a role in limiting TF efficacy in some circumstances. In contrast, thrombin, as an alternate thrombogen, acts later in the cascade, within the common coagulation pathway where the intrinsic and extrinsic pathways merge (Figure 3) [122]. The bypassing of TF and PS requirements through the use of active thrombin theoretically reduces the potential for early feedback inhibition relative to TF and provides a potentially superior effector where increased thrombogenic potential is required, or where cofactors such as PS are not expressed. In the natural cascade, thrombin exists as the pro-enzyme, pro-thrombin, that circulates within the blood stream. Thrombin accumulates at sites of endothelial activation in response to co-expression of thrombin receptors (PARs) and activation of Factor X to Xa. Factor Xa cleaves the pro-thrombin precursor to the active thrombin form, allowing subsequent fibrinogen cleavage and fibrin polymer formation. In addition, several other factors normally work co-operatively to regulate this activation. Anti-thrombogenic molecules that limit thrombin activation typically line the healthy endothelial surface (thrombomodulin/Protein C/Protein S) and patrol the circulation (anti-thrombin III). Thus, although delivery of active thrombin is still subject to inhibiting factors that dictate anti-coagulatory versus pro-coagulatory balance, it does not face the extra restrictions that occur early in the cascade with TF at the endothelial membrane.

Greater potency does come with the caveat of potentially greater risk of disseminated coagulation after systemic infusion if not properly controlled or insufficient care is taken to observe activation thresholds. This may in part explain the limited use of thrombin as an effector relative to tTF. However, limited use may also be due to difficulties with routine and economical production by recombinant approaches, meaning direct conjugation to targeting ligands is cumbersome and poorly reproducible from a clinical standpoint. To date, thrombin use for vascular infarction has been limited to just a handful of studies, where its employment in place of TF serves a specific purpose.

As described earlier, PS-targeting annexin-V coaguligands constructed with thrombin rather than TF were used to induce thrombosis in vitro as well as in vivo in an AVM rat model [40,46]. Thrombin was chosen for these proof-of-principle studies for two primary reasons: (1) early non-targeted studies using soluble, non-targeted TF induced small but not large vessel occlusion in AVMs in vivo, suggesting a more potent thrombogenic effector may be required to occlude large, high flow vessels [36]; and (2) PS-targeting ligands can neutralize TF activity [34]. Gauden et al. demonstrated for the first time that large vessels can be induced to form stable thrombi with targeted thrombin but also that thrombin could be safely administered by systemic infusion within certain dose thresholds [46]. Further studies to assess whether occlusion of large high flow vessels in AVMs could still be achieved with targeted tTF (using surface markers other than PS) are still required to ensure that the safest approach can be taken forward to the clinic.

Alternatively, safety aspects related to thrombin use could be overcome by use of nanotechnology as described by Li et al. in the context of tumor targeting [123]. In this approach, DNA nanobots consisting of sheets of DNA oligonucleotides are connected together by various linkers to form an envelope that encases active thrombin molecules. These thrombin molecules are then delivered safely in an isolated internal environment, separated from the circulation. Upon binding, DNA aptamers, targeting externalized nucleolin on the tumor vasculature, trigger the unfolding of the nanobot, exposing thrombin on the endothelial surface. The nanobots are designed so that aptamers protrude on one side to allow wall attachment, while thrombin molecules become exposed in a unidirectional manner to the lumen and blood flow. Here, the use of thrombin rather than TF is essential, as the nanobots cover the luminal surface and would mask exposure of the TF cofactor, PS, inhibiting VII activation. This approach could be harnessed for use in brain AVMs to overcome any safety concerns regarding the circulation of free active thrombin if suitable targeting ligands or aptamers are developed.

A second study by the latter group also investigated delivery of encapsulated thrombin using chitosan nanoparticles functionalized with the CREKA peptide, targeting fibronectin-fibrin complexes [92]. Moderate tumor thrombosis and inhibition was observed with repeat administration of this construct in breast cancer, melanoma, and liver cancer models, showing thrombin efficacy without off-target effects. However, as with TF use, the most significant results were achieved when DOX was also incorporated in the nanoparticle. Complete tumor regression increased significantly from 20% to 80% with combined thrombin and DOX treatment in the highly vascularized melanoma model.

Whether thrombin can provide a significant advantage over tTF for use in solid tumor infarction remains debatable based on these limited studies. Whether targeted tTF could provide large vessel occlusion as found for thrombin in the context of brain AVMs requires further examination.

### 3.4. Truncated Coagulase (tCoa)

Several ongoing studies have been investigating alternative non-native coaguligands employing a truncated form of the staphylocoagulase produced by *Staphylococcus aureus*, referred to as tCoa [85,86]. This type of coagulase is produced by several pathogenic bacteria and is responsible for the disseminated vascular thrombosis that can occur through the course of sepsis and endocarditis [124]. tCoa acts in the common pathway of the coagulation cascade, as a co-factor of pro-thrombin (Figure 3) [125]. Pro-enzyme binding exerts an allosteric conformational change that forms a ‘staphylothrombin’ complex that allows conversion of fibrinogen to fibrin without the need for prior cleavage of thrombin by the Xa/Va pro-thrombinase complex (Figure 3) [126,127]. Uniquely, this interaction is specific for the conversion of fibrinogen to fibrin. In the normal cascade, activated thrombin also cleaves and activates factors XI, V, and VIII, further amplifying the primary thrombogenic signals (Figure 3). Staphylothrombin does not cleave these pro-enzymes to induce this amplifying effect, however it is also not subject to inhibition and feedback by native thrombin inhibitors (e.g., anti-thrombin III, heparin) [128]. The low minimum dose of 10^−16^ M coagulase estimated to be required for thrombin activation compared to 10^−6^ M for TF and the demonstration of tumor infarction at very low tCoa doses (10–15 µg) suggests this target specificity has an overall positive effect on thrombogenic activity relative to thrombin [73,75,129].

To date, tCoa coaguligands targeting RGD have demonstrated substantial tumor infarction and growth inhibition in colon, breast, and ovarian cancer models [86]. tCoa coaguligands targeting NGR demonstrated similar outcomes in mammary and prostate mouse models [85]. The use of tCoa constructs in these studies did not cause significant disseminated coagulation, as found in septicaemia, leading the authors to suggest the need for co-aggregation to achieve a minimum effective threshold [86], and that in septicaemia this threshold may be exceeded through the formation of bacterial clusters [130]. While studies to date with tCoa are currently limited, this thrombogen theoretically has the potential to increase efficacy over and above that of tTF. Seidi et al. demonstrated that tCoa has a wide therapeutic window, with doses up to 100 µg delivered without toxicity, while earlier reports suggest that TF has an upper dose range of approximately 35 µg [85,90]. Jahanban-Esfahlan et al. postulated that this enzyme may exhibit even greater activity in human blood which is more sensitive to coagulase than mouse blood [114]. Pre-testing of tCoa constructs in parallel-plate flow systems circulating *human* blood over *human* endothelial cell layers may be helpful in this assessment [40,104].

One question remaining regarding tCoa use is whether clots formed are stable and enduring. This is particularly important to consider for the occlusion of larger vessels in AVMs. Activated thrombin in the normal cascade also cleaves the fibrin-stabilizing factor XIII to XIIIa, a plasma transglutaminase essential for fibrin polymer cross-linking and clot stability. Recent in vitro studies demonstrated, however, that staphylothrombin has limited ability to activate XIII relative to thrombin, and produces porous clots with lowered shear stress resistance and accelerated lysis by tissue plasminogen activator (tPA) [131]. Similar thrombin-like serine proteases, found in snake venom, that do not activate XIII also demonstrate similar characteristics and the formation of loose fibrin clots that are rapidly cleared [132]. Studies with tCoa in tumor models did not note inherent clot instability, which may reflect the more complex in vivo milieu and the simultaneous presence of native thrombin. However, it must be carefully examined whether the inability of tCoa to activate the fibrin-stabilizing factor has a significant effect on the risk of clot fragmentation and the potential production of emboli, particularly in the context of large vessel occlusion in AVMs.

One advantage of using tCoa as an effector is that, similar to tTF, it lends itself to the generation of recombinant fusion proteins which make it a clinically viable option for economical production and scale-up. Jahanban-Esfahlan et al. created an active recombinant tCoa protein by producing an N-terminal cleavage site that exposes the Ile-Val residues essential for exosite binding and prothrombin activation [86]. A potential disadvantage may be safety concerns aroused by the bacterial origins of tCoa, however, early studies examining coagulase reactivity suggest antigenicity is not significant [133]. Overall, tCoa appears a viable thrombogen for further comparison in both tumor and AVM models, but it will be important to assess clot stability. In addition, while animal studies provide important proof-of-principle for efficacy of an approach methodologically, important species-specific differences in components of the coagulation cascade must be carefully considered from a safety and toxicity perspective across all animal models at the translational stage, as noted by Berdel et al. in response to recent disparities found between animal models and humans in Phase I studies with tTF [134].

## 4. Conclusions and Future Directions

As an approach to tumor ablation, vascular infarction promises a method to induce rapid tumor resolution with a good therapeutic index relative to other treatment modalities, and may address the issue of drug resistance in chemotherapy and immunotherapy. The literature demonstrates that specific tumor targeting is achievable with currently identified biomarkers in vascular infarction studies, although the heterogeneous nature of the pre-clinical animal studies performed to date makes direct comparisons of different targets difficult. The NGR homing peptide linked to tTF has been advanced furthest and shows significant potential for tumor targeting. While efficacy in humans remains unproven at this stage, altering the progression of disease and outcomes in the advanced cancer patients participating in the early trials was unlikely, and does not necessarily indicate a lack of efficacy. Observations of reduced tumor perfusion with the tTF-NGR construct are, in fact, promising. While some minor thrombotic complications in the early Phase I trials were observed, these events were rectifiable and not of a severity to prevent further human studies. The low number of human trials to date reflects the necessary caution with which the field approaches use of this technique.

Other targets and ligands show potential in pre-clinical studies, and approaches to enhance efficacy such as dual targeting, combinatorial therapies, and composite or nanoparticle delivery, show merit and remain active areas of pre-clinical investigation. To reduce the primary risk of disseminated coagulation, these approaches must minimize the concentration of free circulating thrombogen and enhance local thrombogenic activity at the diseased vessels. In particular, combination therapy with doxorubicin appears highly effective in this context. Targeted delivery of encapsulated thrombogens exposed only at the target site seems the most promising approach to achieve this objective long term, but the nanoparticles themselves must be assessed for safety and tolerability in humans. Further investigation of thrombin and tCoa use in coaguligands is required to assess their relative efficacy compared to tTF and to build confidence in their safety from both medical professionals and patients.

Vascular infarction in AVMs does not suffer the same limitations as tumor infarction, as there is no need to induce cell death; however, complete occlusion of all AVM vessels is still essential for cure. The challenge is ensuring that the thrombosis induced can occlude significantly larger vessels, but also remain stable under high flow. Preliminary studies show that this is achievable using thrombin as an effector, however further work is required to establish the best biomarkers for targeting and the most effective and safe coaguligands in this context. Compared to tumor targeting, this field remains in its infancy, but many lessons can be learned from the advances made in the cancer field. For those 30% of AVM patients that are currently considered untreatable using current methodologies, this approach would provide the first non-invasive pharmacological alternative to cure, and the elimination of the high stroke risk that these young patients face each day.

## Supplementary Materials

The following are available online at https://www.mdpi.com/article/10.3390/biomedicines9070776/s1, Table S1: Studies examining vascular targeting using infarction models.
