# Supplementary material for: The Evolution of Safe and Effective Coaguligands for Vascular Targeting and Precision Thrombosis of Solid Tumors and Vascular Malformations
Source: Biomedicines. 2021 Jul 4;9(7):776. doi: 10.3390/biomedicines9070776 (PMC8301394; doi:10.3390/biomedicines9070776)
Supplement: Supplementary file 1 [file biomedicines-09-00776-s001.zip › biomedicines-1271975-SI.pdf]

Supplementary Material

The evolution of safe and effective coaguligands for vascular targeting and precision thrombosis of solid tumors and vascular malformations

Table S1. Studies examining vascular targeting using infarction models.

Target	Model [(a)]	Targeting Construct	Outcome	Reference
		**Mixed Surface Receptors or Translocated Phospholipids**		
**MHCII (IFN-γ-induced)**	C1300(Muγ) mouse neuroblastoma	Antibody-tTF (0.6 mg/kg) + IFN-γ	Thrombosis, complete tumor regression 38% of animals; volume reduction 70% Day 21	[1]
**Prostate-specific marker antigen**	Rat Mat Lu prostate tumor	PSMA catalytic site inhibitor-tTF (0.1 mg/kg)	Microvessel infarction, tumor growth inhibition 30% Day 7.	[2]
**VCAM-1**	L540rec human Hodgkin's lymphoma	Antibody-tTF (20 μg)	Selective thrombosis; tumor inhibition: 45% Day 21	[3]
**VCAM-1**	L540rec human Hodgkin's lymphoma<br>Colo677 human small cell lung cancer (SCLC) ± human vasculature/HDMEC	Antibody-sTF (20 μg) ± doxorubicin (DOX, 8 mg/kg)	Short term: tumor necrosis, 74% for L540rec + lipopolysaccharide, 26% for Colo677.<br>Long term: delayed tumor growth, 30% Colo677 (Day 14), with DOX 90%	[4]
**Nucleolin**	MDA-MB-231 human breast cancer<br>SK-OV3 human ovarian cancer<br>B16-F10 murine melanoma	Aptamer-guided DNA nanobot (1.5U thrombin/ mouse)	Intravascular thrombosis, growth reduction, tumor necrosis<br>MDA-MB-231: 77% Day 21<br>B16-F10: 67% Day 14<br>SK-OV3:60% Day 21	[5]
**PS**	Vascular injury model (mouse) (not tumor infarction model)	sTF-Annexin V (90 μg/kg)	At low concentrations accelerated blood coagulation, while at higher concentrations acted as an anticoagulant	[6]
**PS**	Rat AVM model	LPS and sTF (non-liganded) ± radiation	Selective small vessel thrombosis observed	[7]
**PS**	Rat AVM model	LPS and sTF (non-liganded) ± radiation	Selective small vessel thrombosis 70% vessels, durable 90 days. Occasional occlusion in large vessels.	[8]
**PS**	Rat AVM model	Annexin V-thrombin protein conjugate ± radiation	Angiographic occlusion in 80% animals after radiation (0.38 mg/kg, single or 2 repeat doses) compared to 0-13% without radiation.	[9]
		**VEGF Receptors**		
**Complex (C6S, VEGFR2, NRP-1)**	N202 murine mammary carcinoma	tTF–truncated heparin binding domain (HBD) (0.5 μg × 2)	Rapid thrombosis, tumor inhibition: 80% with 0.25 μg FVIIa co-administration.	[10]
**VEGFR-1&2**	CT26 murine colorectal cancer	rVEGF-TF (20 μg × 2)	Rapid thrombosis, tumor growth inhibition 87% Day 14.	[11]

Target	Model [(a)]	Targeting Construct	Outcome	Reference
**VEGFR-1**	SMMC-7721 human hepatocarcinoma	SP5.2/tTF OCMCs- SPIO-NPs (0.2 mL, 5 mg Fe/mL)	Conjugated embolic nanoparticle thrombosis and vessel occlusion observed > SP5.2-tTF alone.	[12]
**VEGFR-1**	S180 murine sarcoma	SP5.2-tTF (100 μg)	Thrombosis, growth inhibition/regression 70% Day 6.	[13]
**NRP-1**	HepG2 human liver cancer	Composite system: Antibody -streptavidin + tTF-biotin	Thrombosis, tumor growth reduction and necrosis: 80% Day 7	[14]
**NRP-1**	HepG2 human liver cancer	tTF-EG3287-Cy5.5/chitosan-coated iron oxide NPs (100 μg × 4) + magnetic field	Dual peptide and magnetic field targeted thrombosis and necrosis. Tumor growth reduction: 68% Day 7.	[15]
**NRP-1**	HT29 human colon adenocarcinoma	tTF-EG3287-Cy5.5 iron oxide NPs (as above) + magnetic field	Thrombosis, tumour growth regression: 20% Day 6, 75% Day 27	[16]
**NRP-1**	HepG2 human liver cancer	Composite system: Antibody -streptavidin + tTF-biotin	Thrombosis, tumor growth reduction and necrosis: 80% Day 7	[17]
**NRP-1**	HepG2 human liver cancer	tTF-EG3287 iron oxide NPs (50 μg × 4) × magnetic field	Thrombosis, necrosis, tumor growth inhibition: tTF-EG3287 25% (day 7) tTF-EG3287-NP 75% (day 7)	[18]
		**External Matrix Targets**		
**Fibronectin**	F9 murine teratocarcinoma C51 murine colon carcinoma FE8 rat fibroblast sarcoma	scFv-tTF scFv-tTF (14–35 μg)	High dose, complete tumor regression in 30%, rapid occlusion (1h) in 50%. C51:70% Day 10 (14 μg) F9:45% Day 7 (14 μg) FE8: 75% Day 7 (35 μg)	[19]
**Fibronectin Degenerated vasculature (exposed DNA)**	MAD109 murine lung carcinoma Colon-26 murine colon adenocarcinoma	chimeric antibody –tTF fusions (chTNT-3-tTF, chTV-1-tTF) (3–5 doses, 2.5–40 μg)	Thrombosis and tumor growth inhibition. MAD109: chTNT-3-tTF (2.5–5 μg) 50% Day 19; chTV-1 (20–40 μg) 60% Day 19. Colon-26: chTNT-3-tTF (2.5–10 μg) 25% Day 21; chTNT-3 (40 ug) 55% Day 21 Colon-26 combination therapy: chTNT-3/tTF (2.5 μg) +chTV-1/tTF (20 μg) + RGD/tTF (5 μg); 70% growth inhibition day 21	[20]
**Acidic tumor matrix**	MDA-MB-231 human breast cancer	tTF-pHLIP (20 μg × 4 doses)	Thrombosis, rapid tumor regression: 90% Day 25	[21]

Target	Model (a)	Targeting Construct	Outcome	Reference
**NG2 proteo-glycan (pericytes)**	A549 human lung adenocarcinoma<br>M21 human melanoma	tTF-TAA<br>(0.5–1 mg/kg repeat injections)	Lower anti-tumor activity of TFT-TAA relative to tTF-NGR, smaller therapeutic window. A549: 25% Day 22 (0.5 mg/kg); 40% Day 10 (1 mg/kg). M21: 30% Day 22 (0.5 mg/kg)	[22]
**Fibrin-fibronectin complexes**	LS174T human liver cancer<br>4T1 human breast cancer<br>MHCC97H human liver cancer	tTF-CREKA<br>(2.5 μg)	Thrombosis, tumor growth inhibition<br>4T1: 65% Day15<br>MHCC97H: 60% Day 14<br>LS174T: 47% Day 14	[23]
**Acidic tumor matrix**	B16-F10 murine melanoma	tTF-pHLIP<br>(2.5–5 μg)	Tumor growth inhibition<br>80% Day 9	[24]
**Fibrin-fibronectin complexes**	MDA-MB-231 human breast cancer<br>B16-F10 murine melanoma MHCC97H liver cancer (mouse and rabbit models)	CREKA-Thrombin-DOX-NPs<br>(2.30 U thrombin, chitosan NPs × 6)	MDA-slowed tumor growth, prolonged survival, incomplete blockade.<br>B16-remission without recurrence (day 45): Th-NP 80%; Th-DOX-NP 100% Day 12. MHCC97H-some regrowth: Th-NP 55%; Th-DOX-NP 75% Day12	[25]
		**Integrins or Integrin Receptors**		
**α2bβ3 (ionizing-radiation induced)**	B16F0 murine melanoma	Fibrinogen-coated albumin NPs (embolic) + radiation (3–10 Gy)	Reduced perfusion, tumor growth delay 75% Day 10	[26]
**αvβ3 (CD51)/ α5β1 (CD61)**	CT26 murine colorectal cancer	4 x fibronectin type III motifs-tTF<br>(20 μg)	Localization and local microthrombosis observed histologically immediately after delivery, no growth effects monitored	[27]
**αvβ3**	A549 human lung adenocarcinoma<br>M21 human melanoma HT1080 human fibrosarcoma	tTF-RGD<br>(30 μg)	Tumour growth retardation<br>thrombotic occlusion of tumor vessels<br>A549: 80% Day 26<br>HT1080: 44% Day 7<br>M21: 50% Day 7	[28]
**CD13 and αvβ3**	MDA-MB-435 breast cancer<br>M21 human melanoma	tTF-RGD<br>or tTF-NGR (cyclic peptide fusions–30 μg × 4–5)	Thrombosis induction, tumor growth inhibition:<br>MDA-MB-435:<br>tTF-RGD 42% Day 7<br>tTF-NGR 46% Day 7<br>M21: tTF-NGR7 27% Day 7<br>M21: tTF-NGR8 19% Day 7	[29]
**CD13**	A549 human lung adenocarcinoma<br>M21 human melanoma HT1080 human fibrosarcoma<br>+ Low dosage clinical study (First-in-man, 5 terminal cancer patients–cholangiocarcinoma,	tTF-NGR<br>(30 μg doses-animal models)<br>(1–4 mg/m^2 weekly-human studies)	Animal studies: Vascular tumor volume reduction, regression at high s.c. but some toxicity:<br>A549: 75% Day 26 (30 μg × 6 i.v.)<br>M21: 40% Day 7 (30 μg × 5 i.v.)<br>HT1080: 50% Day 6 (30 μg × 3 i.v.)<br>HT1080: 30% Day 21 (3–5 mg/kg s.c.)	[30]

Target	Model [(a)]	Targeting Construct	Outcome	Reference
	Metastic lung adenocarcinoma, mesothelioma, multiple myeloma, metastatic germ cell tumor)		Long term HT1080: 25% Day 45 (1 mg/kg × 4 i.v.)<br>Long term HT1080: 50% Day 51 (3–5 mg/kg × 2 s.c.)<br>Human studies: 1h i.v. infusion 1–4 mg/m^2 weekly dosing showed good tolerability, no side effects, reduced perfusion metastatic lesions	
**TEM8**	HT-29 human colorectal carcinoma	scFV-tTF (murine TF)	Localized thrombosis, growth inhibition<br>anti-TEM8-tTF: 55% Day 30	[31]
**CD13 and αvβ3**	SKBR3 human breast cancer<br>A549 human lung adenocarcinoma	tTF-NGR & tTF-RGD fusion proteins	Thrombosis, fibrin accumulation, growth inhibition<br>SKBR3: i.v. injections (4 × 1 mg/kg)<br>>50% inhibition Day 9 both tTF-NGR/RGD<br>A549: s.c. injections (3 × 3 mg/kg)<br>>50% inhibition Day 13 tTF-NGR	[32]
**CD13**	HT1080 human fibrosarcoma	tTF-NGR<br>(s.c. 3, 5 or 7 mg/kg)	Tumor growth delay, flow reduction, thrombotic occlusion by s.c. delivery but some<br>toxicity observed (pulmonary embolism, disseminated coagulation, skin bleeding).	[33]
**CD13**	MDA-MB-435 human breast cancer	PEGylated-Gold nanorods/NIR or tTF-RGD<br>(1 mg/kg) +<br>FXIII-LPs	Composite (2-step) system:<br>Thrombosis induced targeted with FXIII-targeted liposomes (LPs) containing DOX<br>(2 mg/kg), 40 × increase drug accumulation, 80% inhibition Day 20.	[34]
**αvβ3**	CT26 murine colorectal cancer	RGD_3-tTF<br>(3 RGD repeat motifs) (50 µg)	Thrombosis and necrosis, tumor growth inhibition 40% Day 10, increased survival (30 day).	[35]
**CD13**	HT1080 human fibrosarcoma<br>A459 human lung adenocarcinoma	TMS(PEG)12-tTF-NGR<br>(1–7 mg/kg)	PEGylated construct decreased activity (perfusion, growth inhibition) but increased LD10, regrowth after drug cessation.<br>HT1080: 30% Day 7 (5 and 7 mg/kg)<br>A549: 20%-38% Day 14 (5 and 7 mg/kg)<br>Therapeutic window: 1/5 mg/kg tTF-NGR; 3 ≥160 for PEGylated tTF-NGR.	[36]
**CD13**	HT1080 human fibrosarcoma<br>U87 human glioblastoma	tTF-NGR<br>(1 mg/kg)	Reduced tumor perfusion observed with multi-modal imaging	[37]
**CD13**	HT1080 human fibrosarcoma	monoPEG-tTF-NGR<br>PEG20k-tTF-NGR	PEGylation reduced efficacy but lower LD10, wider therapeutic window. Growth inhibition 25% Day 28	[38]
**CD13**	HT1080 human fibrosarcoma	tTF-NGR<br>(2 mg/kg i.p. × 3) + low energy ultrasound	Tumor growth inhibition<br>tTF-NGR alone, 40% Day 9<br>tTF-NGR with US, 55% Day 9	[38]

Target	Model (a)	Targeting Construct	Outcome	Reference
**CD13**	M21 human melanoma HT1080 human fibrosarcoma	tTF-NGR or PEGylated tTF-NGR ± DOX	Infarction and complete tumor regression<br>HT1080 at Day 21<br>tTF-NGR (1 mg/kg): 70%<br>PEG-tTFf-NGR (5 mg/kg): 45%<br>DOX + tTF-NGR (5 mg/kg): 90%<br>DOX + PEG-tTf-NGR (5 mg/kg):75%<br>M21 at Day 7<br>tTF-NGR (1.5 mg/kg): 22%<br>DOX + tTF-NGR (5 mg/kg): 26%	[39]
$\alpha v\beta 3$	CT26 murine colon carcinoma<br>4T1 murine breast cancer<br>SKOV3 human ovarian cancer	tCoa-RGD<br>(15 µg)	Rapid selective thrombosis, necrosis<br>4T1: 80% Day 7<br>CT26: 60% Day 7<br>SKOV3: 65% Day 7	[40]
**CD13**	HTB119 small cell lung cancer	tTF-NGR<br>(30 µg × 6)	Tumor growth reduction<br>30% Day 14	[41]
**CD13**	PC3 human prostate cancer<br>4T1 murine breast cancer	tCoa-NGR<br>(10 µg)	Tumor infarction, cell death, growth inhibition. Well tolerated.<br>4T1: 64% Day 7<br>PC3: 53% Day 7	[42]
**CD13**	HT1080 human fibrosarcoma	tTF-NGR<br>(s.c. injection<br>1 mg/kg × 6)	Tumor growth inhibition 30% Day 5 (regrowth after treatment ceased)	[43]
**CD13**	Various models, safety, toxicology and pharmacokinetic studies	tTF-NGR	Various	[44]
**CD13**	Phase I Dose escalation<br>(NCT02902237)<br>17 late stage tumor patients (various)	tTF-NGR	Max tolerable dose (MTD): 3 mg/m^2/day x 5, q day 22<br>Dose limiting toxicity: thromboembolic events, deep vein thrombosis, high sensitivity troponin T elevation	[45]
**CD13**	HT1080 human fibrosarcoma	tTF-NGR<br>(1.5 mg/kg)<br>± radiation	Tumor growth inhibition with tTF-NGR or radiotherapy alone; tumor regression with combined treatment (day 4-10)	[46]

(a) All models are murine unless otherwise stated. Abbreviations: tTF = truncated tissue factor; sTF = soluble tissue factor; AVM = arteriovenous malformation; PS = phosphatidylserine; s.c. = subcutaneous injection; i.v. = intravenous injection; i.p. = intraperitoneal injection; NP = nanoparticle; LPS = lipopolysaccharide

References

1. Huang, X.; Molema, G.; King, S.; Watkins, L.; Edgington, T.S.; Thorpe, P.E. Tumor infarction in mice by antibody-directed targeting of tissue factor to tumor vasculature. *Science* **1997**, *275*, 547–550, doi:10.1126/science.275.5299.547.
2. Liu, C.; Huang, H.; Donate, F.; Dickinson, C.; Santucci, R.; El-Sheikh, A.; Vessella, R.; Edgington, T.S. Prostate-specific membrane antigen directed selective thrombotic infarction of tumors. *Cancer Res.* **2002**, *62*, 5470–5475.
3. Ran, S.; Gao, B.; Duffy, S.; Watkins, L.; Rote, N.; Thorpe, P.E. Infarction of solid Hodgkin's tumors in mice by antibody-directed targeting of tissue factor to tumor vasculature. *Cancer Res.* **1998**, *58*, 4646–4653.
4. Dienst, A.; Grunow, A.; Unruh, M.; Rabausch, B.; Nor, J.E.; Fries, J.W.; Gottstein, C. Specific occlusion of murine and human tumor vasculature by VCAM-1-targeted recombinant fusion proteins. J. Natl. Cancer Inst. 2005, 97, 733–747, doi:10.1093/jnci/dji130.
5. Li, S.; Jiang, Q.; Liu, S.; Zhang, Y.; Tian, Y.; Song, C.; Wang, J.; Zou, Y.; Anderson, G.J.; Han, J.Y.; et al. A DNA nanorobot functions as a cancer therapeutic in response to a molecular trigger in vivo. *Nat. Biotechnol.* **2018**, *36*, 258–264, doi:10.1038/nbt.4071.
6. Huang, X.; Ding, W.Q.; Vaught, J.L.; Wolf, R.F.; Morrissey, J.H.; Harrison, R.G.; Lind, S.E. A soluble tissue factor-annexin V chimeric protein has both procoagulant and anticoagulant properties. *Blood* **2006**, *107*, 980–986, doi:10.1182/blood-2005-07-2733.
7. Storer, K.; Tu, J.; Karunanayaka, A.; Smee, R.; Short, R.; Thorpe, P.; Stoodley, M. Coadministration of low-dose lipopolysaccharide and soluble tissue factor induces thrombosis after radiosurgery in an animal arteriovenous malformation model. Neurosurgery 2007, 61, 604–611, doi:10.1227/01.neu.0000290909.32600.a8.
8. Reddy, R.; Duong, T.T.; Fairhall, J.M.; Smee, R.I.; Stoodley, M.A. Durable thrombosis in a rat model of arteriovenous malformation treated with radiosurgery and vascular targeting. *J. Neurosurg.* **2014**, *120*, 113–119, doi:10.3171/2013.9.jns122056.
9. Gauden, A.J.; McRobb, L.S.; Lee, V.S.; Subramanian, S.; Moutrie, V.; Zhao, Z.; Stoodley, M.A. Occlusion of Animal Model Arteriovenous Malformations Using Vascular Targeting. *Transl. Stroke Res.* **2020**, *11*, 689–699, doi:10.1007/s12975-019-00759-y.
10. El-Sheikh, A.; Borgstrom, P.; Bhattacharjee, G.; Belting, M.; Edgington, T.S. A selective tumor microvasculature thrombogen that targets a novel receptor complex in the tumor angiogenic microenvironment. *Cancer Res.* **2005**, *65*, 11109–11117, doi:10.1158/0008-5472.CAN-05-2733.
11. Huang, F.Y.; Li, Y.N.; Wang, H.; Huang, Y.H.; Lin, Y.Y.; Tan, G.H. A fusion protein containing murine vascular endothelial growth factor and tissue factor induces thrombogenesis and suppression of tumor growth in a colon carcinoma model. *J. Zhejiang Univ. Sci. B* **2008**, *9*, 602–609, doi:10.1631/jzus.B0820120.
12. Chen, X.; Lv, H.; Ye, M.; Wang, S.; Ni, E.; Zeng, F.; Cao, C.; Luo, F.; Yan, J. Novel superparamagnetic iron oxide nanoparticles for tumor embolization application: Preparation, characterization and double targeting. *Int. J. Pharm.* **2012**, *426*, 248–255, doi:10.1016/j.ijpharm.2012.01.043.
13. Lv, S.; Ye, M.; Wang, X.; Li, Z.; Chen, X.; Dou, X.; Dai, Y.; Zeng, F.; Luo, L.; Wang, C.; et al. A recombined fusion protein SP5.2/tTF induce thrombosis in tumor blood vessel. *Neoplasma* **2015**, *62*, 531–540, doi:10.4149/neo_2015_064.
14. Xu, P.; Zou, M.; Wang, S.; Li, T.; Liu, C.; Wang, L.; Wang, L.; Luo, F.; Wu, T.; Yan, J. Construction and characterization of a truncated tissue factorcoagulation based composite system for selective thrombosis in tumor blood vessels. *Int. J. Oncol.* **2019**, *55*, 823–832, doi:10.3892/ijo.2019.4855.
15. Zou, M.; Samiullah, M.; Xu, P.; Wang, S.; He, J.; Wu, T.; Luo, F.; Yan, J. Construction of novel procoagulant protein targeting neuropilin-1 on tumour vasculature for tumour embolization therapy. *J. Drug Target.* **2019**, *27*, 885–895, doi:10.1080/1061186x.2019.1566337.
16. Qiu, G.; Xie, X.; Zhao, B.; Xu, L.; Chen, Y. Fusion protein tTF-EG3287 induces occlusion of tumor vessels and impairs tumor growth in human colon cancer. *Neoplasma* **2019**, *66*, 252–260, doi:10.4149/neo_2018_180722N513.
17. Xu, P.; Zou, M.; Wang, S.; Wang, L.; Wang, L.; Luo, F.; Wu, T.; Yan, J. Preparation of truncated tissue factor antineuropilin-1 monoclonal antibody conjugate and identification of its selective thrombosis in tumor blood vessels. *Anticancer Drugs* **2019**, *30*, 441–450, doi:10.1097/cad.0000000000000767.
18. Zou, M.; Xu, P.; Wang, L.; Wang, L.; Li, T.; Liu, C.; Shi, L.; Xie, J.; Li, W.; Wang, S.; et al. Design and construction of a magnetic targeting pro-coagulant protein for embolic therapy of solid tumors. *Artif. Cells Nanomed. Biotechnol.* **2020**, *48*, 116–128, doi:10.1080/21691401.2019.1699817.
19. Nilsson, F.; Kosmehl, H.; Zardi, L.; Neri, D. Targeted delivery of tissue factor to the ED-B domain of fibronectin, a marker of angiogenesis, mediates the infarction of solid tumors in mice. *Cancer Res.* **2001**, *61*, 711–716.
20. Hu, P.; Yan, J.; Sharifi, J.; Bai, T.; Khawli, L.A.; Epstein, A.L. Comparison of three different targeted tissue factor fusion proteins for inducing tumor vessel thrombosis. *Cancer Res.* **2003**, *63*, 5046–5053.
21. Li, S.; Tian, Y.; Zhao, Y.; Zhang, Y.; Su, S.; Wang, J.; Wu, M.; Shi, Q.; Anderson, G.J.; Thomsen, J.; et al. pHLIP-mediated targeting of truncated tissue factor to tumor vessels causes vascular occlusion and impairs tumor growth. *Oncotarget* **2015**, *6*, 23523–23532, doi:10.18632/oncotarget.4395.
22. Brand, C.; Schliemann, C.; Ring, J.; Kessler, T.; Baumer, S.; Angenendt, L.; Mantke, V.; Ross, R.; Hintelmann, H.; Spieker, T.; et al. NG2 proteoglycan as a pericyte target for anticancer therapy by tumor vessel infarction with retargeted tissue factor. *Oncotarget* **2016**, *7*, 6774–6789, doi:10.18632/oncotarget.6725.
23. Shi, Q.; Zhang, Y.; Liu, S.; Liu, G.; Xu, J.; Zhao, X.; Anderson, G.J.; Nie, G.; Li, S. Specific tissue factor delivery using a tumor-homing peptide for inducing tumor infarction. *Biochem. Pharmacol.* **2018**, *156*, 501–510, doi:10.1016/j.bcp.2018.09.020.

24. Ding, L.; Zhang, C.; Liu, Z.; Huang, Q.; Zhang, Y.; Li, S.; Nie, G.; Tang, H.; Wang, Y. Metabonomic Investigation of Biological Effects of a New Vessel Target Protein tTF-pHLIP in a Mouse Model. *J. Proteome Res.* **2019**, *19*, 238–247, doi:10.1021/acs.jproteome.9b00507.
25. Li, S.; Zhang, Y.; Ho, S.-H.; Li, B.; Wang, M.; Deng, X.; Yang, N.; Liu, G.; Lu, Z.; Xu, J. Combination of tumour-infarction therapy and chemotherapy via the co-delivery of doxorubicin and thrombin encapsulated in tumour-targeted nanoparticles. *Nat. Biomed. Eng.* **2020**, *4*, 732–742, doi:10.1038/s41551-020-0573-2.
26. Hallahan, D.; Geng, L.; Qu, S.; Scarfone, C.; Giorgio, T.; Donnelly, E.; Gao, X.; Clanton, J. Integrin-mediated targeting of drug delivery to irradiated tumor blood vessels. *Cancer Cell* **2003**, *3*, 63–74.
27. Liu, C.; Dickinson, C.; Shobe, J.; Doñate, F.; Ruf, W.; Edgington, T. A hybrid fibronectin motif protein as an integrin targeting selective tumor vascular thrombogen. *Mol. Cancer Ther.* **2004**, *3*, 793–801.
28. Kessler, T.; Bieker, R.; Padro, T.; Schwoppe, C.; Persigehl, T.; Bremer, C.; Kreuter, M.; Berdel, W.E.; Mesters, R.M. Inhibition of tumor growth by RGD peptide-directed delivery of truncated tissue factor to the tumor vasculature. *Clin. Cancer Res.* **2005**, *11*, 6317–6324, doi:10.1158/1078-0432.ccr-05-0389.
29. Kessler, T.; Schwoppe, C.; Liersch, R.; Schliemann, C.; Hintelmann, H.; Bieker, R.; Berdel, W.E.; Mesters, R.M. Generation of fusion proteins for selective occlusion of tumor vessels. *Curr. Drug Discov. Technol.* **2008**, *5*, 1–8, doi:10.2174/157016308783769487.
30. Bieker, R.; Kessler, T.; Schwöppe, C.; Padró, T.; Persigehl, T.; Bremer, C.; Dreischalück, J.; Kolkmeyer, A.; Heindel, W.; Mesters, R.M. Infarction of tumor vessels by NGR-peptide–directed targeting of tissue factor: Experimental results and first-in-man experience. *Blood* **2009**, *113*, 5019–5027, doi:10.1182/blood-2008-04-150318.
31. Fernando, S.; Fletcher, B.S. Targeting tumor endothelial marker 8 in the tumor vasculature of colorectal carcinomas in mice. *Cancer Res.* **2009**, *69*, 5126–5132, doi:10.1158/0008-5472.can-09-0725.
32. Schwoppe, C.; Kessler, T.; Persigehl, T.; Liersch, R.; Hintelmann, H.; Dreischaluck, J.; Ring, J.; Bremer, C.; Heindel, W.; Mesters, R.M.; et al. Tissue-factor fusion proteins induce occlusion of tumor vessels. *Thromb. Res.* **2010**, *125* (Suppl. 2), S143–S150, doi:10.1016/s0049-3848(10)70033-5.
33. Dreischalück, J.; Schwöppe, C.; Spieker, T.; Kessler, T.; Tiemann, K.; Liersch, R.; Schliemann, C.; Kreuter, M.; Kolkmeyer, A.; Hintelmann, H. Vascular infarction by subcutaneous application of tissue factor targeted to tumor vessels with NGR-peptides: Activity and toxicity profile. *Int. J. Oncol.* **2010**, *37*, 1389–1397, doi:10.3892/ijo_00000790.
34. von Maltzahn, G.; Park, J.H.; Lin, K.Y.; Singh, N.; Schwoppe, C.; Mesters, R.; Berdel, W.E.; Ruoslahti, E.; Sailor, M.J.; Bhatia, S.N. Nanoparticles that communicate in vivo to amplify tumour targeting. *Nat. Mater.* **2011**, *10*, 545–552, doi:10.1038/nmat3049.
35. Huang, Z.J.; Zhao, Y.; Luo, W.Y.; You, J.; Li, S.W.; Yi, W.C.; Wang, S.Y.; Yan, J.H.; Luo, Q. Targeting the vasculature of colorectal carcinoma with a fused protein of (RGD)(3)-tTF. *ScientificWorldJournal* **2013**, *2013*, 637086, doi:10.1155/2013/637086.
36. Schwoppe, C.; Zerbst, C.; Frohlich, M.; Schliemann, C.; Kessler, T.; Liersch, R.; Overkamp, L.; Holtmeier, R.; Stypmann, J.; Dreiling, A.; et al. Anticancer therapy by tumor vessel infarction with polyethylene glycol conjugated retargeted tissue factor. *J. Med. Chem.* **2013**, *56*, 2337–2347, doi:10.1021/jm301669z.
37. Persigehl, T.; Ring, J.; Bremer, C.; Heindel, W.; Holtmeier, R.; Stypmann, J.; Claesener, M.; Hermann, S.; Schafers, M.; Zerbst, C.; et al. Non-invasive monitoring of tumor-vessel infarction by retargeted truncated tissue factor tTF-NGR using multi-modal imaging. *Angiogenesis* **2014**, *17*, 235–246, doi:10.1007/s10456-013-9391-4.
38. Brand, C.; Dencks, S.; Schmitz, G.; Mühlmeister, M.; Stypmann, J.; Ross, R.; Hintelmann, H.; Schliemann, C.; Müller-Tidow, C.; Mesters, R.M.; et al. Low-Energy Ultrasound Treatment Improves Regional Tumor Vessel Infarction by Retargeted Tissue Factor. *J. Ultrasound Med.* **2015**, *34*, 1227–1236, doi:10.7863/ultra.34.7.1227.
39. Stucke-Ring, J.; Ronnacker, J.; Brand, C.; Holtke, C.; Schliemann, C.; Kessler, T.; Schmidt, L.H.; Harrach, S.; Mantke, V.; Hintelmann, H.; et al. Combinatorial effects of doxorubicin and retargeted tissue factor by intratumoral entrapment of doxorubicin and proapoptotic increase of tumor vascular infarction. *Oncotarget* **2016**, *7*, 82458–82472, doi:10.18632/oncotarget.12559.
40. Jahanban-Esfahlan, R.; Seidi, K.; Monhemi, H.; Adli, A.D.F.; Minofar, B.; Zare, P.; Farajzadeh, D.; Farajnia, S.; Behzadi, R.; Abbasi, M.M. RGD delivery of truncated coagulase to tumor vasculature affords local thrombotic activity to induce infarction of tumors in mice. *Sci. Rep.* **2017**, *7*, 1–14, doi:10.1038/s41598-017-05326-9.
41. Schmidt, L.H.; Stucke-Ring, J.; Brand, C.; Schliemann, C.; Harrach, S.; Muley, T.; Herpel, E.; Kessler, T.; Mohr, M.; Gorlich, D.; et al. CD13 as target for tissue factor induced tumor vascular infarction in small cell lung cancer. *Lung Cancer* **2017**, *113*, 121–127, doi:10.1016/j.lungcan.2017.09.013.
42. Seidi, K.; Jahanban-Esfahlan, R.; Monhemi, H.; Zare, P.; Minofar, B.; Daei Farshchi Adli, A.; Farajzadeh, D.; Behzadi, R.; Mesgari Abbasi, M.; Neubauer, H.A.; et al. NGR (Asn-Gly-Arg)-targeted delivery of coagulase to tumor vasculature arrests cancer cell growth. *Oncogene* **2018**, *37*, 3967–3980, doi:10.1038/s41388-018-0213-4.
43. Kessler, T.; Baumeier, A.; Brand, C.; Grau, M.; Angenendt, L.; Harrach, S.; Stalmann, U.; Schmidt, L.H.; Gosheger, G.; Hardes, J.; et al. Aminopeptidase N (CD13): Expression, Prognostic Impact, and Use as Therapeutic Target for Tissue Factor Induced Tumor Vascular Infarction in Soft Tissue Sarcoma. *Transl. Oncol.* **2018**, *11*, 1271–1282, doi:10.1016/j.tranon.2018.08.004.
44. Berdel, W.E.; Harrach, S.; Brand, C.; Brömmel, K.; Berdel, A.F.; Hintelmann, H.; Schliemann, C.; Schwöppe, C. Animal Safety, Toxicology, and Pharmacokinetic Studies According to the ICH S9 Guideline for a Novel Fusion Protein tTF-NGR Targeting Procoagulatory Activity into Tumor Vasculature: Are Results Predictive for Humans? *Cancers (Basel)* **2020**, *12*, 3536, doi:10.3390/cancers12123536.
45. Schliemann, C.; Gerwing, M.; Heinzow, H. First-In-Class CD13-Targeted Tissue Factor tTF-NGR in Patients with Recurrent or Refractory Malignant Tumors: Results of a Phase I Dose-Escalation Study. *Cancers (Basel)* **2020**, *12*, 1488, doi:10.3390/cancers12061488.

46. Brand, C.; Greve, B.; Bölling, T.; Eich, H.T.; Willich, N.; Harrach, S.; Hintelmann, H.; Lenz, G.; Mesters, R.M.; Kessler, T.; et al. Radiation synergizes with antitumor activity of CD13-targeted tissue factor in a HT1080 xenograft model of human soft tissue sarcoma. *PLoS ONE* **2020**, *15*, e0229271, doi:10.1371/journal.pone.0229271.
